# Fungal Masquerader: A Case Report of Extramammary Paget’s Disease Treated with Radiotherapy

**DOI:** 10.7759/cureus.5937

**Published:** 2019-10-17

**Authors:** Jason R Mammino, Robert P Daze, Jere Mammino

**Affiliations:** 1 Internal Medicine, Largo Medical Center, Largo, USA; 2 Dermatology, Largo Medical Center, Largo, USA; 3 Dermatology, KCU-GME Consortium/Orlando Dermatology Program, Maitland, USA

**Keywords:** dermatology, extramammary paget's disease, cutaneous neoplasm, superficial electron beam radiotherapy

## Abstract

An 83-year-old Caucasian male presented with a pruritic erythematous patch to his right inguinal region, which he had contracted five months ago. After months of topical antifungal and steroid therapies, the patient was referred to dermatology due to a lack of lesion improvement. A 5.0 mm punch biopsy with immunohistochemical staining revealed the presence of pleomorphic nuclei with cytoplasm replete with mucin, suggestive of superficial extramammary Paget’s disease (EMPD). As he was reluctant to undergo a surgical assessment, the patient underwent consultation and management with 30 sessions of superficial electron beam radiotherapy. A week after the completion of radiation therapy, the patient’s skin exhibited minimal erythema with surrounding hyperpigmentation to the affected inguinal skin, suggesting clearance of the disease. This case highlights the importance of an accurate diagnosis in a timely manner as neoplastic cases have a metastatic risk with potentially devastating results.

## Introduction

Extramammary Paget’s disease (EMPD) is a rare intraepithelial neoplastic dermatosis. Only a few hundred documented cases have been reported in the literature with the exact number of incidences unknown [1]. This disease is most commonly seen in elderly Caucasian females, with vulvar tissue being the area that is affected in the most number of people [2,3]. EMPD within the perineal or perianal tissue has the highest association with internal malignancy [4]. With an untrained eye, the time to diagnosis may be extended up to two years or more, which could worsen the prognosis [1]. In our study, we report a case of EMPD and discuss the clinical presentation, histopathology, various management types, and common differentials for cutaneous manifestations of genital and perianal cutaneous conditions.

## Case presentation

**Figure 1:**
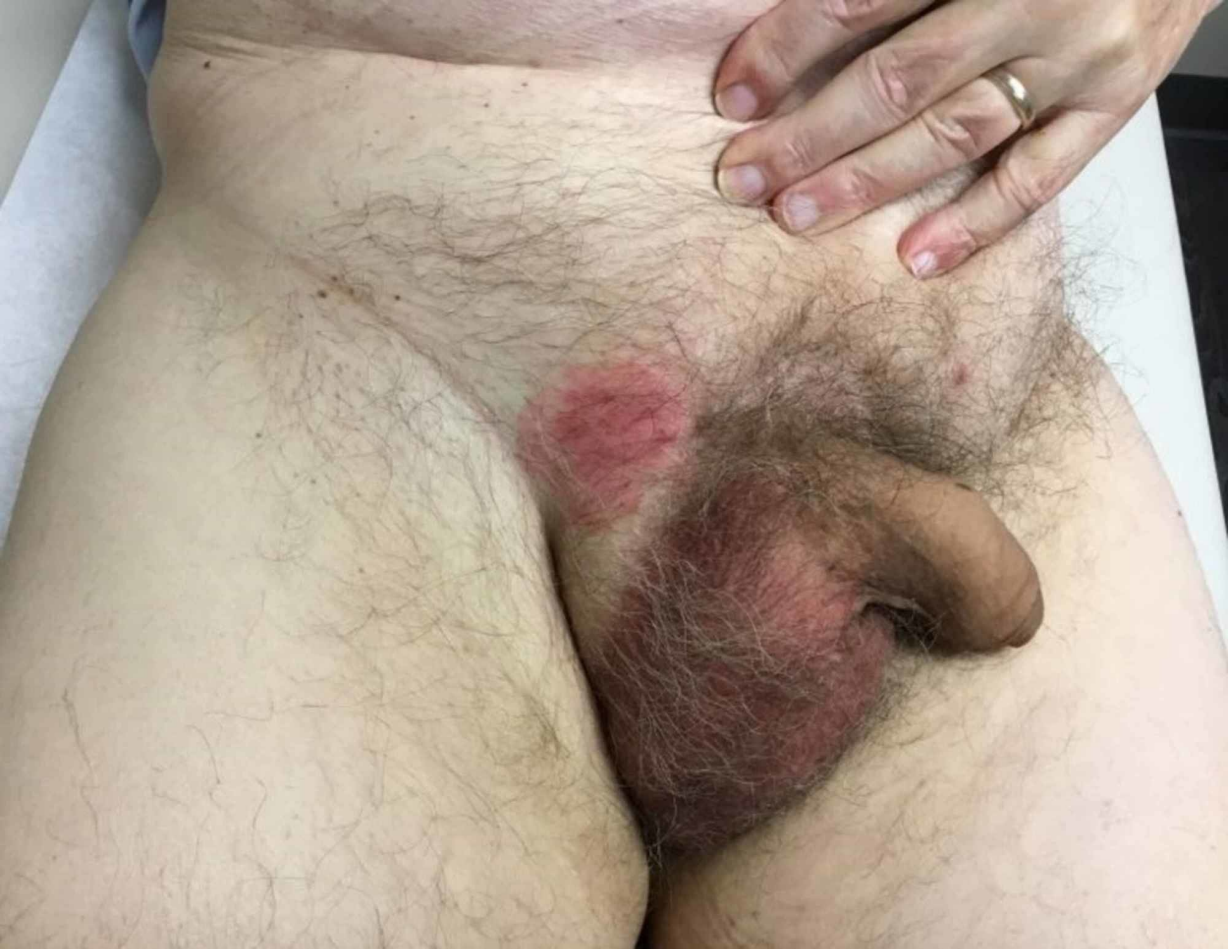
Disease presentation before radiotherapy, which shows a unilateral 5.0 cm erythematous patch to the right inguinal region devoid of scaling

An 83-year-old Caucasian male with a past medical history of essential hypertension, hyperlipidemia, stage-3 chronic kidney disease, benign prostate hyperplasia, and type 2 diabetes mellitus presented with a five-month-old recalcitrant pruritic erythematous patch on his right inguinal skin (Figure 1).

**Figure 2:**
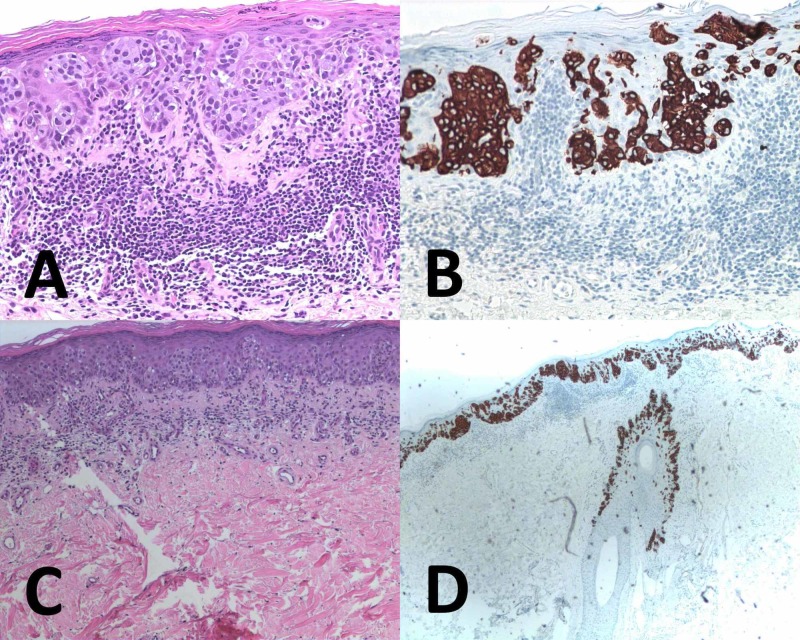
Right inguinal skin immunohistochemical stains A and C: hematoxylin and eosin stain, high and low power. Intraepidermal nests of amphophilic cytoplasm exhibiting a typical pagetoid appearance and a crushed basal layer with underlying lymphocytic infiltrate. B and D: cytokeratin 7 stain, high and low power. Highlighting of the tumor cells can be visualized within the epidermis and adjacent follicle

On initial presentation to his primary-care physician, a diagnosis of dermatitis was suspected. He was prescribed a steroid cream, which he applied twice daily to the affected area. After three months, the patient’s rash remained unchanged and the diagnosis was shifted to a presumed fungal infection. Following two months of twice-a-day use of ketoconazole cream, the patient’s rash continued to exhibit no signs of lesion improvement, prompting the primary-care physician to refer him to dermatology. With the persistent history of failed topical therapies, the patient subsequently underwent a punch biopsy to his right inguinal region. Immunohistochemical staining of 5.0 mm punched tissue highlighted the presence of pleomorphic nuclei with cytoplasm replete with mucin, suggestive of superficial EMPD (Figure 2).

Both sry-related HMg-Box 10 (SOX-10) and transformation-related protein 63 (p63) stains failed to show any evidence of melanocytic/neural lesions or evidence of carcinoma, respectively.

The patient was informed of the diagnosis and given options for further management, including surgical excision and radiation therapy. The patient ultimately decided on pursuing a radio-therapeutic approach and was seen by his radiation consultation within the month. With the risk of underlying internal malignancy, the patient underwent colonoscopy as well as a positron emission tomography (PET) scan. No polypoid lesions or increased lymphovascular uptake were seen on either diagnostic modality. With his history of prostate growth, the patient’s prostate-specific antigen (PSA) level was found to be less than four. A targeted approach employing a generous margin of radiation of 60 Gy at 1.8-2 Gy per day for a total of 30-33 treatments was designed. Throughout a six-week course, the patient was managed with a total of 6,000 cGy in 30 fractions of superficial electron beam radiotherapy by way of radiation oncology.

**Figure 3:**
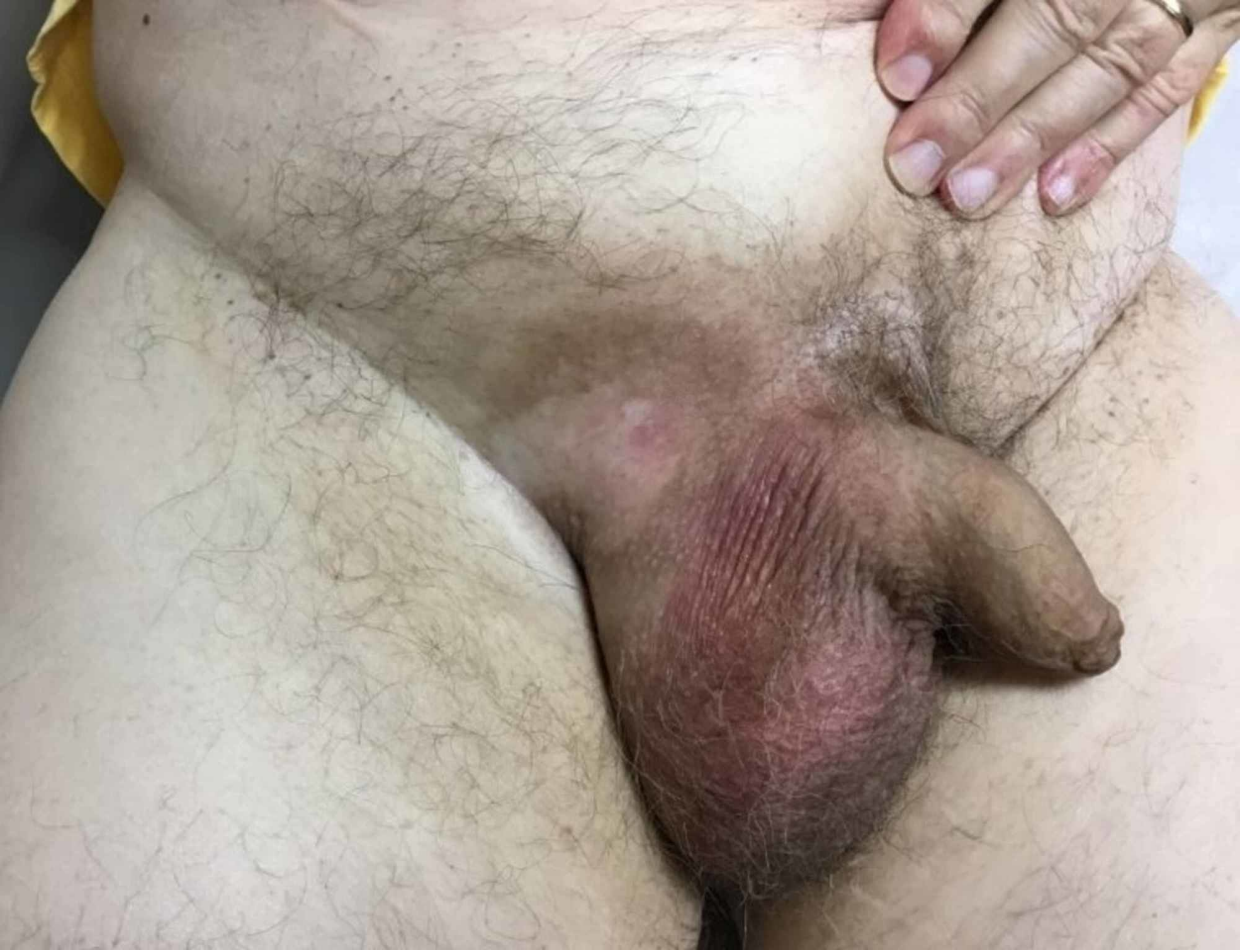
Disease presentation after radiotherapy, which shows a glabrous pink patch with central hypopigmentation with surrounding post-inflammatory hyperpigmentation to the right inguinal region

Examination with dermatology a week after the completion of radiotherapy showed minimal erythema, with central hypopigmentation and surrounding hyperpigmentation to the affected inguinal skin (Figure 3). No signs of active disease were determined. The patient had scheduled follow-up appointments with the oncologist at the first and fourth month, as well as with his dermatologist at the eighth month, post-radiation completion. All his follow-up examinations were uneventful and continued to show no signs of disease recurrence. Due to the high recurrence rates of EMPD, close follow-ups involving six-month-to-yearly skin examinations were recommended with a low threshold for additional biopsies.

## Discussion

EMPD is a rare neoplastic dermatosis with metastatic potential [2,4,5]. Currently, it is observed to occur in two forms. Primary or superficial EMPD originates within the epidermis or apocrine glands and is confined to the epidermal layer [2,4,5]. With similarities seen between Toker cells, which are found in a small percentage of nipple tissues, and EMPD, their presence might possibly be a precursor to developing EMPD [2,6]. Primary disease is sometimes referred to as an in situ type of cancer due to its low potential for metastasis [4,5]. Secondary EMPD is a less common form and usually develops due to an underlying carcinoma. Cutaneous disease is triggered by the epidermotropic spread of malignant cells from an adnexal or visceral tumor in close proximity [4,5]. There is an extremely rare third type of EMPD where the epidermis is devoid of apocrine glands. This type is called ectopic EMPD, and a few such cases have documented the involvement of areas such as the abdomen or eyelid [7].

The most common presentation of EMPD occurs in the vulvar tissue of Caucasian females, while the perianal disease type has mostly been reported in Japanese males [2,4]. One typically will observe a slowly expanding erythematous plaque that can have associated erosions with scattered white plaques, giving the classic "strawberries and cream" appearance [2].

For an accurate diagnosis, immunohistochemical stains are of great value. Common features of EMPD acknowledged under hematoxylin and eosin stain are large cells within the epidermis exhibiting amphophilic cytoplasm, intraepidermal nests (buckshot scatter), or a crushed basilar layer [8]. Cytokeratin (CK) 7 and CK 20 are such stains that can help differentiate between primary and secondary EMPD. Tumors with CK 7 positive and CK 20 negative are usually indicative of primary disease, whereas those with CK 7 negative and CK 20 positive usually indicate secondary disease [2,5]. Both primary and secondary EMPD patients should undergo a thorough investigation for internal malignancy. Pelvis ultrasound, CT of the abdomen and pelvis, and PET scans along with endoscopic exams are crucial [4,5].

There are a variety of treatments available for the management of EMPD. However, due to multifocal growth with subclinical expansion into normal-appearing adjacent tissue, recurrence can occur in 20-40% of the cases [2,4]. The most common modality is surgical excision [5]. The original method of wide local excision is slowly being replaced with Mohs micrographic surgery due to decreased recurrence rates associated with the latter [5]. With the difficulty in determining malignant margins, this technique maximizes tissue sparing while minimizing the surgical defect. Other options include topical and systemic chemotherapy, photodynamic therapy, laser therapy, radiation therapy, or a combination of all sorts [4,5]. The prognosis for superficial disease without metastasis is often very good. Unfortunately, if the lymphovascular system is compromised, the five-year survival is 0% [4,5]. 

In our case, considering the absence of any lymphatic involvement, superficial appearance, the advanced age of the patient, and his reluctance to undergo surgical excision, radiotherapy was selected as the mode of treatment. The patient should continue to undergo close follow-up and skin exams as even a PET scan with no evidence of lymphatic spread can give only a limited understanding regarding the potential for any future metastasis.

## Conclusions

When examining a rash for the first time, the importance of accurate morphologic descriptions cannot be overstated as diseases such as tinea have characteristic visual features. Common dermatoses to the groin are classically infectious or inflammatory in etiology and can have secondary characteristics such as scale or crust. Tinea cruris, allergic and irritant contact dermatitis, intertrigo, candidal infections, seborrheic dermatitis, and psoriasis all fall under these categories. With regard to our patient, having minimal response to topical antifungal and steroid application, the differential was expanded to include the neoplastic realm. Bowen’s disease, parapsoriasis, mycosis fungoides, and EMPD were all hypothesized given his lesional resistance. Having a persistent lesion despite standard-of-care therapies should prompt clinicians to explore a histologic approach with consideration for referral to dermatology. Being familiar with morphologic appearances of common skin manifestations to the genital and perianal regions can lead to accurate and timely diagnoses, earlier treatments, and superior patient outcomes in the treatment of EMPD.
